# 4-Amino-3-[(4-methoxy­phen­yl)amino­meth­yl]-1*H*-1,2,4-triazole-5(4*H*)-thione

**DOI:** 10.1107/S1600536809032607

**Published:** 2009-08-22

**Authors:** Hoong-Kun Fun, Chin Sing Yeap, Shridhar Malladi, Mahesh Padaki, Arun M. Isloor

**Affiliations:** a X-ray Crystallography Unit, School of Physics, Universiti Sains Malaysia, 11800 USM, Penang, Malaysia; b Department of Chemistry, National Institute of Technology-Karnataka, Surathkal, Mangalore 575 025, India

## Abstract

The mol­ecule of the title compound, C_10_H_13_N_5_OS, is approximately planar, the dihedral angle between the triazole and benzene rings being 4.53 (10)°. The amino group adopts a pyramidal configuration. In the crystal structure, mol­ecules are linked into two-dimensional networks parallel to (001) by inter­molecular N—H⋯S and N—H⋯N hydrogen bonds. In addition, an S⋯S short contact of 3.3435 (7) Å is observed.

## Related literature

For the pharmacological applications of 1,2,4-triazole derivatives, see: Amir *et al.* (2008 ▶); Isloor *et al.* (2009 ▶); Krzysztof *et al.* (2008 ▶); Kuş *et al.* (2008 ▶); Padmavathi *et al.* (2008 ▶). For the preparation, see: Holla & Udupa (1992 ▶). For related structures, see: Fun *et al.* (2009*a* ▶,*b* ▶). For the stability of the temperature controller used for data collection, see: Cosier & Glazer (1986 ▶).
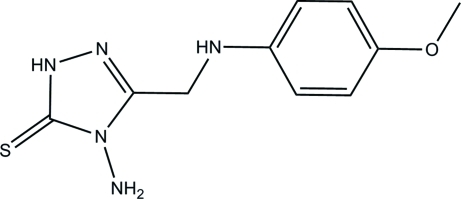


## Experimental

### 

#### Crystal data

C_10_H_13_N_5_OS
*M* *_r_* = 251.31
Monoclinic, 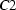

*a* = 11.5142 (2) Å
*b* = 5.8804 (1) Å
*c* = 16.6891 (3) Å
β = 95.292 (1)°
*V* = 1125.17 (3) Å^3^
*Z* = 4
Mo *K*α radiation
μ = 0.28 mm^−1^
*T* = 100 K
0.33 × 0.18 × 0.02 mm

#### Data collection

Bruker SMART APEXII CCD area-detector diffractometer
Absorption correction: multi-scan (**SADABS**; Bruker, 2005 ▶) *T*_min_ = 0.913, *T*_max_ = 0.994
5827 measured reflections
2365 independent reflections
2126 reflections with *I* > 2σ(*I*)
*R*_int_ = 0.024

#### Refinement

*R*[*F* ^2^ > 2σ(*F* ^2^)] = 0.031
*wR*(*F* ^2^) = 0.068
*S* = 1.04
2365 reflections
171 parameters
1 restraint
H atoms treated by a mixture of independent and constrained refinement
Δρ_max_ = 0.33 e Å^−3^
Δρ_min_ = −0.26 e Å^−3^
Absolute structure: Flack (1983 ▶), 588 Friedel pairs
Flack parameter: 0.03 (7)

### 

Data collection: *APEX2* (Bruker, 2005 ▶); cell refinement: *SAINT* (Bruker, 2005 ▶); data reduction: *SAINT*; program(s) used to solve structure: *SHELXTL* (Sheldrick, 2008 ▶); program(s) used to refine structure: *SHELXTL*; molecular graphics: *SHELXTL*; software used to prepare material for publication: *SHELXTL* and *PLATON* (Spek, 2009 ▶).

## Supplementary Material

Crystal structure: contains datablocks global, I. DOI: 10.1107/S1600536809032607/ci2889sup1.cif

Structure factors: contains datablocks I. DOI: 10.1107/S1600536809032607/ci2889Isup2.hkl

Additional supplementary materials:  crystallographic information; 3D view; checkCIF report

## Figures and Tables

**Table 1:** Hydrogen-bond geometry (Å, °)

*D*—H⋯*A*	*D*—H	H⋯*A*	*D*⋯*A*	*D*—H⋯*A*
N2—H1*N*2⋯S1^i^	0.84 (2)	2.55 (2)	3.3672 (18)	164 (2)
N4—H1*N*4⋯N5^ii^	0.82 (3)	2.60 (3)	3.410 (2)	169 (3)
N5—H1*N*5⋯N1^iii^	0.89 (2)	2.48 (2)	3.133 (2)	130 (2)

Symmetry codes: (i) 
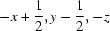
; (ii) 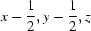; (iii) 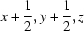.

**Fig. 1.**
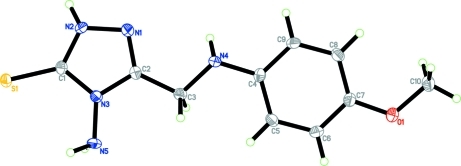
The molecular structure of the title compound, with atom labels and 50% probability ellipsoids for non-H atoms.

**Fig. 2.**
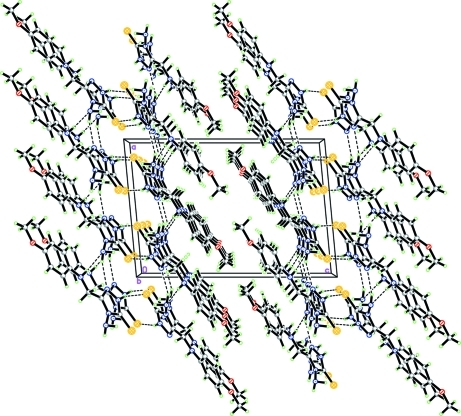
The crystal structure of the title compound, viewed down the b axis, showing two-dimensional networks parallel to ab plane. Intermolecular hydrogen bonds are shown as dashed lines.

## supplementary crystallographic information

### Comment

1,2,4-triazole and its derivatives were reported to exhibit various pharmacological activities such as antimicrobial, analgesic, anti-inflammatory, anticancer and antioxidant properties (Amir *et al.*, 2008; Krzysztof *et al.*, 2008; Kuş *et al.*, 2008; Padmavathi *et al.*, 2008). A few derivatives of triazoles have exhibited antimicrobial activity (Isloor *et al.*, 2009). Some of the present day drugs such as ribavirin (antiviral agent), rizatriptan (antimigraine agent), alprazolam (anxiolytic agent), fluconazole and itraconazole (antifungal agents) are the best examples for potent molecules possessing the triazole nucleus. The amino and mercapto groups of 1,2,4-triazoles serve as readily accessible nucleophilic centers for the preparation of N-bridged heterocycles. Keeping in view of the biological importance, we have synthesized the title compound and its crystal structure is reported here.

Bond lengths and angles in the title compound (Fig. 1) are comparable to those observed in related structures (Fun *et al.*, 2009*a,b*). The molecule is approximately planar, with the dihedral angle between the triazole (N1/N2/C1/N3/C2) and benzene rings (C4-C9) being 4.53 (10)°.

In the crystal structure, the molecules are linked by intermolecular N2—H1N2···S1, N4—H1N4···N5 and N5—H1N5···N1 hydrogen bonds into a two-dimensional network parallel to *ab* plane (Fig. 2 and Table 1). In addition, a S···S(1-x, y, -z) short contact of 3.3435 (7) Å is observed.

### Experimental

2-[(4-Methoxyphenyl)amino]acetohydrazide (19.5 g, 0.1 mol) was added slowly to a solution of potassium hydroxide (8.4 g, 0.15 mol) in ethanol (150 ml). The resulting mixture was stirred well till a clear solution was obtained. Carbon disulfide (11.4 g, 0.15 mol) was added dropwise and the contents were stirred vigorously. Further stirring was continued for 24 h. The resulting mixture (Holla & Udupa, 1992) was diluted with 100 ml of ether and the precipitate formed was collected by filtration, washed with dry ether and dried at 338 K under vacuum. It was used for the next step without any purification.

A mixture of above synthesized potassium dithiocarbazinate (30.9 g, 0.1 mol), hydrazine hydrate (99%, 0.2 mol) and water (2 ml) was gently heated to boil for 30 min. Heating was continued until the evaluation of hydrogen sulfide ceases. The reaction mixture was cooled to room temperature, diluted with water (100 ml) and acidified with 2 N HCl. The solid mass that separated was collected by filtration, washed with water and dried. Recrystallization was done from ethanol (yield: 15.1 g, 67.7%; m.p. 484–486 K).

### Refinement

N-bound H atoms were located in a difference Fourier map and refined freely. The remaining H atoms were positioned geometrically [C-H = 0.93–0.97 Å] and refined using a riding model, with *U*_iso_(H) = 1.2*U*_eq_(C) and 1.5*U*_eq_(methyl C). A rotating group model was used for the methyl groups.

### Crystal data

**Table:**

C_10_H_13_N_5_OS	*F*(000) = 528
*M**_r_* = 251.31	*D*_x_ = 1.484 Mg m^−^^3^
Monoclinic, *C*2	Mo *K*α radiation, λ = 0.71073 Å
Hall symbol: C 2y	Cell parameters from 2961 reflections
*a* = 11.5142 (2) Å	θ = 3.6–32.2°
*b* = 5.8804 (1) Å	µ = 0.28 mm^−^^1^
*c* = 16.6891 (3) Å	*T* = 100 K
β = 95.292 (1)°	Plate, colourless
*V* = 1125.17 (3) Å^3^	0.33 × 0.18 × 0.02 mm
*Z* = 4	

### Data collection

**Table:**

Bruker SMART APEXII CCD area-detector diffractometer	2365 independent reflections
Radiation source: fine-focus sealed tube	2126 reflections with *I* > 2σ(*I*)
graphite	*R*_int_ = 0.024
φ and ω scans	θ_max_ = 30.0°, θ_min_ = 3.6°
Absorption correction: multi-scan (*SADABS*; Bruker, 2005)	*h* = −15→16
*T*_min_ = 0.913, *T*_max_ = 0.994	*k* = −8→5
5827 measured reflections	*l* = −23→23

### Refinement

**Table:**

Refinement on *F*^2^	Secondary atom site location: difference Fourier map
Least-squares matrix: full	Hydrogen site location: inferred from neighbouring sites
*R*[*F*^2^ > 2σ(*F*^2^)] = 0.031	H atoms treated by a mixture of independent and constrained refinement
*wR*(*F*^2^) = 0.068	*w* = 1/[σ^2^(*F*_o_^2^) + (0.0277*P*)^2^ + 0.6764*P*] where *P* = (*F*_o_^2^ + 2*F*_c_^2^)/3
*S* = 1.04	(Δ/σ)_max_ = 0.001
2365 reflections	Δρ_max_ = 0.33 e Å^−^^3^
171 parameters	Δρ_min_ = −0.25 e Å^−^^3^
1 restraint	Absolute structure: Flack (1983), 588 Friedel pairs
Primary atom site location: structure-invariant direct methods	Flack parameter: 0.03 (7)

### Special details

**Table:**

Experimental. The crystal was placed in the cold stream of an Oxford Cyrosystems Cobra open-flow nitrogen cryostat (Cosier & Glazer, 1986) operating at 100.0 (1) K.
Geometry. All e.s.d.'s (except the e.s.d. in the dihedral angle between two l.s. planes) are estimated using the full covariance matrix. The cell e.s.d.'s are taken into account individually in the estimation of e.s.d.'s in distances, angles and torsion angles; correlations between e.s.d.'s in cell parameters are only used when they are defined by crystal symmetry. An approximate (isotropic) treatment of cell e.s.d.'s is used for estimating e.s.d.'s involving l.s. planes.
Refinement. Refinement of *F*^2^ against ALL reflections. The weighted *R*-factor *wR* and goodness of fit *S* are based on *F*^2^, conventional *R*-factors *R* are based on *F*, with *F* set to zero for negative *F*^2^. The threshold expression of *F*^2^ > σ(*F*^2^) is used only for calculating *R*-factors(gt) *etc*. and is not relevant to the choice of reflections for refinement. *R*-factors based on *F*^2^ are statistically about twice as large as those based on *F*, and *R*- factors based on ALL data will be even larger.

### Fractional atomic coordinates and isotropic or equivalent isotropic displacement parameters (Å^2^)

**Table:**

	*x*	*y*	*z*	*U*_iso_*/*U*_eq_	
S1	0.37035 (4)	0.34412 (9)	0.03759 (3)	0.01571 (11)	
O1	−0.26796 (11)	1.2919 (3)	0.42305 (8)	0.0248 (4)	
N1	0.07536 (13)	0.3929 (3)	0.13658 (9)	0.0171 (4)	
N2	0.15394 (14)	0.2872 (3)	0.09067 (10)	0.0164 (4)	
N3	0.23563 (13)	0.5947 (3)	0.13167 (9)	0.0136 (3)	
N4	−0.02128 (14)	0.6826 (3)	0.24608 (10)	0.0167 (4)	
N5	0.30984 (14)	0.7845 (3)	0.14247 (10)	0.0171 (4)	
C1	0.25184 (15)	0.4074 (3)	0.08580 (11)	0.0144 (4)	
C2	0.12844 (15)	0.5803 (4)	0.16083 (11)	0.0138 (4)	
C3	0.08129 (16)	0.7617 (4)	0.21027 (12)	0.0158 (4)	
H3A	0.1404	0.8072	0.2524	0.019*	
H3B	0.0613	0.8933	0.1768	0.019*	
C4	−0.08548 (14)	0.8413 (4)	0.28707 (10)	0.0146 (3)	
C5	−0.04122 (16)	1.0556 (4)	0.31036 (12)	0.0174 (4)	
H5A	0.0307	1.1019	0.2950	0.021*	
C6	−0.10392 (16)	1.1985 (4)	0.35610 (12)	0.0187 (4)	
H6A	−0.0732	1.3398	0.3715	0.022*	
C7	−0.21184 (16)	1.1350 (4)	0.37941 (11)	0.0168 (4)	
C8	−0.25711 (16)	0.9225 (4)	0.35578 (11)	0.0174 (4)	
H8A	−0.3295	0.8776	0.3708	0.021*	
C9	−0.19487 (15)	0.7792 (4)	0.31039 (11)	0.0163 (4)	
H9A	−0.2261	0.6384	0.2949	0.020*	
C10	−0.37125 (17)	1.2205 (4)	0.45705 (12)	0.0219 (5)	
H10A	−0.3982	1.3406	0.4896	0.033*	
H10B	−0.3545	1.0881	0.4897	0.033*	
H10C	−0.4305	1.1848	0.4147	0.033*	
H1N2	0.1335 (18)	0.175 (4)	0.0617 (13)	0.016 (6)*	
H1N4	−0.063 (2)	0.601 (5)	0.2156 (15)	0.032 (7)*	
H1N5	0.381 (2)	0.728 (5)	0.1550 (16)	0.047 (8)*	
H2N5	0.3141 (19)	0.845 (6)	0.0921 (15)	0.042 (7)*	

### Atomic displacement parameters (Å^2^)

**Table:**

	*U*^11^	*U*^22^	*U*^33^	*U*^12^	*U*^13^	*U*^23^
S1	0.01344 (18)	0.0157 (2)	0.0184 (2)	0.0019 (2)	0.00344 (15)	−0.0002 (2)
O1	0.0220 (7)	0.0201 (9)	0.0342 (8)	−0.0015 (6)	0.0123 (6)	−0.0096 (7)
N1	0.0157 (7)	0.0161 (10)	0.0201 (8)	−0.0007 (7)	0.0052 (6)	0.0000 (7)
N2	0.0186 (7)	0.0117 (9)	0.0197 (8)	−0.0021 (6)	0.0050 (6)	−0.0030 (7)
N3	0.0126 (7)	0.0149 (9)	0.0135 (7)	−0.0008 (6)	0.0015 (6)	−0.0003 (7)
N4	0.0150 (8)	0.0149 (9)	0.0208 (9)	−0.0039 (7)	0.0051 (7)	−0.0033 (7)
N5	0.0142 (7)	0.0148 (9)	0.0220 (9)	−0.0050 (6)	0.0006 (6)	−0.0019 (7)
C1	0.0143 (8)	0.0155 (10)	0.0135 (8)	0.0025 (7)	0.0011 (7)	0.0001 (7)
C2	0.0132 (8)	0.0156 (10)	0.0126 (9)	0.0022 (7)	0.0015 (7)	0.0029 (8)
C3	0.0151 (8)	0.0140 (10)	0.0183 (9)	−0.0012 (7)	0.0020 (7)	0.0006 (8)
C4	0.0153 (7)	0.0154 (9)	0.0132 (7)	0.0032 (9)	0.0012 (6)	0.0006 (10)
C5	0.0139 (9)	0.0176 (11)	0.0211 (10)	−0.0015 (8)	0.0043 (7)	0.0005 (8)
C6	0.0188 (9)	0.0146 (10)	0.0228 (10)	−0.0027 (8)	0.0020 (8)	−0.0037 (8)
C7	0.0174 (9)	0.0165 (10)	0.0167 (9)	0.0015 (8)	0.0030 (7)	−0.0005 (8)
C8	0.0157 (9)	0.0184 (10)	0.0187 (9)	−0.0014 (8)	0.0044 (7)	0.0013 (8)
C9	0.0163 (8)	0.0121 (10)	0.0203 (9)	−0.0024 (7)	0.0002 (7)	−0.0006 (8)
C10	0.0177 (9)	0.0258 (13)	0.0228 (10)	0.0016 (9)	0.0057 (8)	−0.0032 (9)

### Geometric parameters (Å, °)

**Table:**

S1—C1	1.6883 (18)	C3—H3A	0.97
O1—C7	1.373 (2)	C3—H3B	0.97
O1—C10	1.427 (2)	C4—C5	1.401 (3)
N1—C2	1.306 (3)	C4—C9	1.401 (2)
N1—N2	1.386 (2)	C5—C6	1.383 (3)
N2—C1	1.339 (2)	C5—H5A	0.93
N2—H1N2	0.84 (2)	C6—C7	1.387 (3)
N3—C1	1.364 (3)	C6—H6A	0.93
N3—C2	1.370 (2)	C7—C8	1.397 (3)
N3—N5	1.407 (2)	C8—C9	1.377 (3)
N4—C4	1.407 (3)	C8—H8A	0.93
N4—C3	1.448 (2)	C9—H9A	0.93
N4—H1N4	0.82 (3)	C10—H10A	0.96
N5—H1N5	0.89 (3)	C10—H10B	0.96
N5—H2N5	0.92 (3)	C10—H10C	0.96
C2—C3	1.482 (3)		
			
C7—O1—C10	117.60 (17)	H3A—C3—H3B	108.1
C2—N1—N2	103.83 (15)	C5—C4—C9	118.09 (18)
C1—N2—N1	113.14 (16)	C5—C4—N4	122.53 (16)
C1—N2—H1N2	125.0 (14)	C9—C4—N4	119.3 (2)
N1—N2—H1N2	120.6 (14)	C6—C5—C4	120.33 (17)
C1—N3—C2	108.88 (16)	C6—C5—H5A	119.8
C1—N3—N5	126.84 (15)	C4—C5—H5A	119.8
C2—N3—N5	124.04 (17)	C5—C6—C7	121.20 (19)
C4—N4—C3	118.22 (18)	C5—C6—H6A	119.4
C4—N4—H1N4	112.7 (18)	C7—C6—H6A	119.4
C3—N4—H1N4	112.5 (17)	O1—C7—C6	116.46 (18)
N3—N5—H1N5	105.8 (19)	O1—C7—C8	124.69 (17)
N3—N5—H2N5	105.9 (18)	C6—C7—C8	118.83 (18)
H1N5—N5—H2N5	103 (2)	C9—C8—C7	120.19 (17)
N2—C1—N3	103.45 (15)	C9—C8—H8A	119.9
N2—C1—S1	129.38 (15)	C7—C8—H8A	119.9
N3—C1—S1	127.15 (15)	C8—C9—C4	121.4 (2)
N1—C2—N3	110.68 (18)	C8—C9—H9A	119.3
N1—C2—C3	126.50 (17)	C4—C9—H9A	119.3
N3—C2—C3	122.78 (18)	O1—C10—H10A	109.5
N4—C3—C2	110.69 (17)	O1—C10—H10B	109.5
N4—C3—H3A	109.5	H10A—C10—H10B	109.5
C2—C3—H3A	109.5	O1—C10—H10C	109.5
N4—C3—H3B	109.5	H10A—C10—H10C	109.5
C2—C3—H3B	109.5	H10B—C10—H10C	109.5
			
C2—N1—N2—C1	−0.9 (2)	N3—C2—C3—N4	−168.35 (17)
N1—N2—C1—N3	1.1 (2)	C3—N4—C4—C5	−14.9 (3)
N1—N2—C1—S1	179.40 (14)	C3—N4—C4—C9	169.36 (17)
C2—N3—C1—N2	−0.9 (2)	C9—C4—C5—C6	1.0 (3)
N5—N3—C1—N2	−175.43 (17)	N4—C4—C5—C6	−174.84 (19)
C2—N3—C1—S1	−179.25 (15)	C4—C5—C6—C7	−0.5 (3)
N5—N3—C1—S1	6.3 (3)	C10—O1—C7—C6	−171.73 (18)
N2—N1—C2—N3	0.2 (2)	C10—O1—C7—C8	10.0 (3)
N2—N1—C2—C3	177.94 (18)	C5—C6—C7—O1	−178.46 (18)
C1—N3—C2—N1	0.4 (2)	C5—C6—C7—C8	−0.1 (3)
N5—N3—C2—N1	175.13 (17)	O1—C7—C8—C9	178.47 (18)
C1—N3—C2—C3	−177.35 (17)	C6—C7—C8—C9	0.2 (3)
N5—N3—C2—C3	−2.7 (3)	C7—C8—C9—C4	0.2 (3)
C4—N4—C3—C2	−172.48 (15)	C5—C4—C9—C8	−0.8 (3)
N1—C2—C3—N4	14.2 (3)	N4—C4—C9—C8	175.13 (18)

### Hydrogen-bond geometry (Å, °)

**Table:**

*D*—H···*A*	*D*—H	H···*A*	*D*···*A*	*D*—H···*A*
N2—H1N2···S1^i^	0.84 (2)	2.55 (2)	3.3672 (18)	164 (2)
N4—H1N4···N5^ii^	0.82 (3)	2.60 (3)	3.410 (2)	169 (3)
N5—H1N5···N1^iii^	0.89 (2)	2.48 (2)	3.133 (2)	130 (2)

Symmetry codes: (i) −*x*+1/2, *y*−1/2, −*z*; (ii) *x*−1/2, *y*−1/2, *z*; (iii) *x*+1/2, *y*+1/2, *z*.
